# Management of Bulky Tumors in Cervical Cancer: Limits of the Surgical Approach

**DOI:** 10.3390/jcm14041142

**Published:** 2025-02-10

**Authors:** María Alonso-Espías, Fátima Pérez, Myriam Gracia, Ignacio Zapardiel

**Affiliations:** 1 Gynecologic Oncology Unit, La Paz University Hospital, Paseo de la Castellana 261, 28046 Madrid, Spain; 2 Gynecology and Obstetrics, Rafael Méndez University Hospital, Ctra N-340, 30813 Lorca, Spain

**Keywords:** cervical cancer, bulky tumors, neoadjuvant chemotherapy, adjuvant radiotherapy

## Abstract

The standard treatment for cervical tumors larger than 4 cm, known as bulky tumors, currently involves concurrent chemoradiotherapy followed by vaginal brachytherapy. However, radical surgery is an alternative option in some cases, particularly for those in which a combination of risk factors is not anticipated. Recent studies show that neoadjuvant chemotherapy may help reduce tumor size in these bulky tumors, enabling subsequent surgical intervention reducing the adverse effects derived from radiotherapy. Evidence about fertility sparing surgery in patients with bulky tumors is limited, although some retrospective studies reported good oncological outcomes when adequate tumor reduction is achieved through neoadjuvant chemotherapy. Moreover, the administration of adjuvant radiotherapy after radical surgery in patients with tumor sizes ≥ 4 cm in the final pathological report, combined with other intermediate risk factors for recurrence, remains a topic of debate. Current evidence indicates no significant differences in overall survival or disease-free survival between follow-up alone and the use of adjuvant radiotherapy in these cases, although further research is needed to refine treatment strategies for these patients. This narrative review aims to summarize the available evidence on the comprehensive management of bulky cervical tumors, addressing relevant issues and controversies in the field.

## 1. Introduction

Cervical cancer is the fourth most frequent malignancy and the fourth leading cause of cancer-related death among women worldwide [1]. Treatment strategies are determined by the tumor stage at diagnosis. For early-stage disease, surgery remains the treatment of choice, with radical hysterectomy with pelvic lymph node assessment being the standard management. In these stages, radiotherapy (RT) has shown similar survival rates but is associated with more side effects. For locally advanced stages, however, the standard approach is concurrent chemoradiotherapy (CTRT) followed by vaginal brachytherapy (BT). The combination of surgery and RT is associated with an increased morbidity and should be avoided [2].

**Figure 1:**
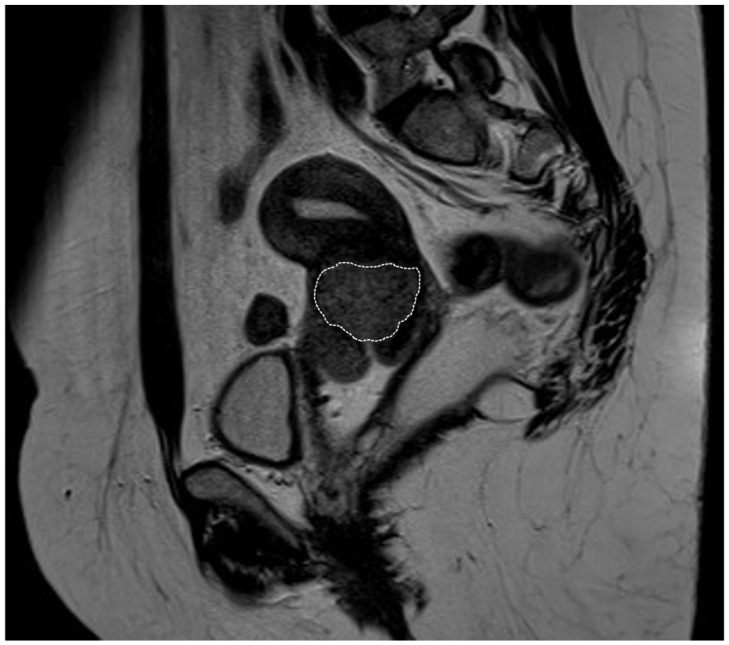
Pelvic magnetic resonance imaging of a bulky cervical tumor (marked with the dotted circle). The image corresponds to a 43-year-old patient diagnosed with cervical squamous cell carcinoma, with tumor dimensions of 43 × 41 × 39 mm on imaging and no parametrial involvement. She underwent primary surgical treatment with bilateral pelvic lymphadenectomy and radical hysterectomy (in 2015), with a postoperative stage of IIA2 (FIGO 2018). Adjuvant treatment with external beam radiotherapy (EBRT), chemotherapy (CT), and brachytherapy (BT) was administered. She was disease-free at the time of writing this article.

Bulky cervical tumors are defined as those with a diameter equal or greater than 4 cm (Figure 1), corresponding to stages IB3 and IIA2 according to the International Federation of Gynecology and Obstetrics (FIGO) staging classification of 2018 [3]. Since most patients with large tumor sizes commonly associate parametrial invasion or lymph node involvement, only 10% of cervical cancer patients are diagnosed at either of these two stages, resulting in scarce evidence on the most appropriate management in these specific cases [2]. Patients with bulky tumors are more likely to present other high-risk factors for recurrence at the final pathological study such as lymph node involvement, parametrial invasion, or positive surgical resection margins, presenting, therefore, greater probability to require adjuvant CTRT after surgery. For this reason, the standard treatment for these patients according to the European and the National Comprehensive Cancer Network (NCCN) guidelines is concurrent CTRT followed by BT. However, there is controversy on the most appropriate treatment modality for these patients, and type C2 radical surgery, after negative pelvic lymph node evaluation, is also allowed as an alternative option [2,4].

Furthermore, it should be noted that these patients already present one of the intermediate recurrence risk criteria described by Sedlis et al. in 1999, so they are also more likely to receive adjuvant RT after surgery if they also present lymphovascular space invasion (LVSI) or deep stromal invasion, leading to a combination of treatments that result in an increased morbidity [5]. Despite this, as the Sedlis study was published two decades ago and the surgical selection criteria used at the time may lack the precision of current standards, there is currently great controversy about the benefit of adjuvant treatment in such patients.

In addition, recent studies highlight the possible benefits of neoadjuvant chemotherapy (NACT) in reducing tumor volume in patients with bulky cervical tumors [6]. In cases achieving partial or complete response, this approach facilitates subsequent surgery while minimizing the adverse effects associated with pelvic RT, which is especially relevant for younger patients, who are the most commonly diagnosed with these tumors [7]. Moreover, some studies have evaluated the possibility of performing fertility sparing surgery (FSS) after NACT if a good response was achieved, although limited data are available, and it should not be recommended outside clinical trials [8].

The objective of this narrative review is to analyze and synthesize the current evidence on the management of patients with bulky cervical tumors. To conduct this review, we searched various databases, including PubMed, for studies addressing this topic. Given the limited literature available, we did not restrict our search to a specific time frame; however, we primarily focused on publications from the past ten years.

## 2. Update Perspective on Bulky Cervical Tumors

### 2.1. Role of Surgery in Bulky Tumors

As mentioned before, surgery is the standard treatment for early-stage cervical cancer, but the surgical approach for locally bulky cervical tumors remains unclear. Tumors with a size equal or greater than 4 cm are associated with worse survival and greater local pelvic recurrence regardless of treatment modality [9].

For early bulky tumors (stages IB3 and IIA2 according to FIGO 2018 classification), treatment options include radical surgery (radical hysterectomy with pelvic lymphadenectomy) with or without adjuvant therapy, primary radiation, or primary concurrent CTRT followed by vaginal BT. However, the optimal treatment is still under debate [10].

Radical surgery offers several advantages over RT. It allows the analysis of pathological characteristics and provides more precise staging, which can guide the selection of postoperative adjuvant therapies and gives prognostic information. Additionally, in young patients, surgery can reduce damage to ovarian function and prevent complications such as vaginal fibrosis and vaginal fistula caused by BT. Consequently, patients tend to experience a better quality of life following radical surgery compared to RT [11,12,13].

**Figure 2:**
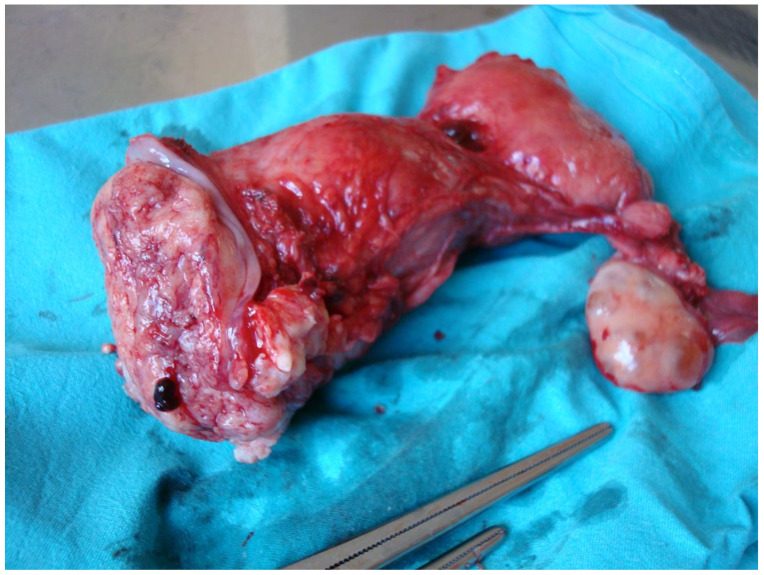
Surgical specimen of a cervical bulky tumor after cut-through surgery. The image corresponds to a 39-year-old patient diagnosed with a 44 × 40 cm cervical squamous cell carcinoma. The preoperative assessment showed no evidence of parametrial invasion. However, during the radical hysterectomy procedure, the left parametrium appeared to be involved, which was later confirmed by the histopathological analysis of the surgical specimen. Despite receiving adjuvant CTRT, the patient experienced a recurrence at 14 months and ultimately passed away due to disease progression.

Surgical treatment for this type of tumor, after a negative assessment of the pelvic lymph nodes, aims to remove the primary cervical lesion along with the surrounding tissue that may be affected, thus reducing the risk of local recurrence and distant metastasis (Figure 2). Some authors suggest a type C2 radical hysterectomy without nerve preservation to ensure a complete resection of the ventral, lateral, and dorsal parametria in three dimensions, as the standard treatment for bulky tumors [14].

For the assessment of lymph node involvement, computed tomography (CT) and magnetic resonance imaging (MRI) are associated with a high false-negative rate, ranging from 20% to 50%. Positron emission tomography–computed tomography (PET-CT), however, offers superior detection rates, although with widely variable sensitivity reported in the literature, ranging from 36% to 86%. Therefore, in the subgroup of patients with negative PET-CT, performing para-aortic lymphadenectomy is considered particularly useful. When lymph node metastases smaller than 5 mm are detected (in approximately 12% of cases), extending the irradiation field achieves survival rates comparable to those of node-negative patients who do not undergo extended field treatment. Furthermore, in patients with negative PET findings, tumor size is an additional factor to consider when indicating para-aortic lymphadenectomy, as approximately 21% of such cases present with positive lymph nodes [15,16,17,18,19].

In addition to tumor size, other clinical prognostic factors such as tumor markers levels and tumor histology, significantly influence treatment response and survival [9]. Numerous studies have demonstrated that adenocarcinoma (AC) and adenosquamous carcinoma (ASC) histologies negatively affect clinical outcomes in patients undergoing radiation therapy, primarily due to the radioresistant nature of these tumor subtypes [20,21,22]. However, there are currently no differences in treatment between the different histological subtypes [2,4]. On the other hand, in terms of tumor markers, a high level of CEA in patients with squamous cell carcinoma (SCC) receiving concurrent CTRT is associated with local failure and worse disease-free survival (DFS). Chen et al. conducted a study published in 2023 with the objective of evaluating the difference in clinical outcomes in 685 patients grouped according to their different prognoses between radical surgery and concurrent chemoradiation as primary treatment. Patients with stage IB2 or IIA2 (according to 2009 FIGO staging system) and tumor histologies of SCC, AC, or ASC were included in the study. A high-risk group was identified, comprising patients with SC and CEA levels ≥ 10 ng/mL or those with AC or ASC histologies, while a low-risk group included patients who did not meet these criteria. In the surgery group, primary treatment included radical hysterectomy with pelvic and/or paraaortic lymphadenectomy. RT or CTRT was administered after surgery for the intermediate-risk group and the high-risk group, respectively, according to the pathological risk factors. In the high-risk group, they found that primary concurrent CTRT caused approximately 15% more local failure and less DFS compared with primary surgery group. On the other hand, locoregional recurrence and DFS of CTRT were comparable to those achieved with radical surgery in patients without risk factors. The study concluded that in early-stage cervical cancer patients with large tumors and high-risk features, primary radical surgery, with or without adjuvant treatment, provides superior DFS and local control, while in patients without high-risk characteristics, both radical surgery with or without adjuvant radiation and primary concurrent CTRT represent effective treatment options [9].

Other published studies such as that of Huang et al. showed that, in patients with AC or ASC histologies, radical hysterectomy resulted in better 5-year DFS compared to primary RT/CTRT for bulky IB-IIA cervical tumors (91% vs. 48% respectively) [21,23]. These studies suggest the possibility that the radioresistant nature of AC and ASC could be overcome by surgery, and RS should be considered as the primary treatment for these bulky AC/ASC tumors.

In 2020, Liu et al. reported that the 5-year overall survival (OS) rates for radical surgery were better than those for primary concurrent CTRT in an analysis of long-term oncological outcomes of patients with cervical cancer stage IB2 to IIA2 of 37 Chinese hospitals (81.5% vs. 72.5%) [24]. Rungrugang et al. also demonstrated that treatment with radical surgery in patients with early-stage bulky cervical tumors was better than primary treatment with RT in terms of OS and specific survival [25]. Along the same lines, Landoni et al. reported a lower recurrence rate in patients with early-stage bulky tumors treated with radical surgery compared with the recurrence rate of patients treated with RT (34% vs. 42%). These results show the limitations of RT in this type of tumors [26].

Zhou et al. published a retrospective study that analyzed survival, complications, and prognostic factors in 975 patients with early-stage bulky cervical cancer (IB2 or IIA2 according to the 2009 FIGO staging system) who underwent radical surgery with or without adjuvant therapy [27]. Radical surgery included Querleu–Morrow type C2 radical hysterectomy and bilateral pelvic lymph node dissection, which encompassed the common, internal, and external iliac lymph nodes; deep inguinal lymph nodes; and obturator lymph nodes. Bilateral common iliac lymph nodes were subjected to intraoperative assessment. Para-aortic abdominal lymph node dissection was indicated if enlarged para-aortic lymph nodes were detected during intraoperative examination or if common iliac lymph nodes were positive during intraoperative assessment. After surgery, patients with high-risk factors, including positive lymph nodes, parametrial involvement, and positive resection margins, received adjuvant CTRT. Those with two or more intermediate risk factors, including positive LVSI, deep stromal invasion, and tumor diameter > 4 cm, received adjuvant CTRT/RT. According to the results obtained, the 5-year DFS was 80.8% and the 5-year OS was 85.9%. Univariate analysis showed that FIGO stage, tumor diameter, histological subtype, parametrial involvement, nodal involvement, deep stromal invasion, and LVSI were associated with lower DFS and OS (*p* < 0.05). The 5-year DFS and OS rates for SCC were significantly higher than the rates for AC/ASC. Regarding the pelvic recurrence rate, patients treated with radical surgery and adjuvant treatment had a significantly lower recurrence rate compared to those treated with radical surgery alone (5.0% vs. 11.3%) [27].

Regarding the most optimal surgical approach for the treatment of this type of tumor, there are few published studies available. Liu et al. conducted a study in 2019 including 39 patients with histologically confirmed bulky cervical cancer (IB3, IIA2, and IIB stages according to the 2018 FIGO staging system) treated with NACT followed by radical hysterectomy. In 18 patients, the intervention was performed robotically, while in 21 patients, it was conducted through conventional abdominal surgery. Their results showed that patients treated with robotic surgery had better perioperative outcomes but not better survival outcomes. Furthermore, the robotic approach was associated with higher recurrence and mortality rates [28]. However, the available data are limited, and further studies are needed to draw definitive conclusions. Table 1 summarizes the main characteristics and survival outcomes of the available evidence regarding radical hysterectomy in patients with bulky tumors.

### 2.2. Neoadjuvant Chemotherapy in Bulky Tumors Before Surgery

As previously discussed, although it is not the standard treatment, NACT is one of the possible therapeutic options for patients with bulky tumors [10].

In resource-limited settings, implementing the CTRT regimen can be challenging due to financial constraints and a lack of chemotherapy (CT) administration and radiation delivery systems, such as linear accelerators and BT equipment. NACT provides an opportunity for early systemic treatment of micrometastases and allows for the assessment of tumor response to CT. It can also enable radical hysterectomy with negative surgical margins, reduce RT toxicity, and compensate for the scarcity of radiotherapy machines or expertise. Other theoretical advantages of NACT, whether delivered prior to radical surgery or radical RT, have been demonstrated in clinical practice through improved survival rates. This is attributed to a reduction in distant failure rates; decreased local failure (due to higher resectability with negative margins or improved radiation efficacy through better oxygenation and reduced exposure of surrounding normal tissues as a result of tumor volume reduction); and enhanced organ preservation, such as ovarian function. Additionally, by minimizing RT toxicity, NACT may facilitate fertility preservation without compromising oncological outcomes [29]. However, this approach may yield negative outcomes in cases of suboptimal cisplatin doses or prolonged intervals between therapy cycles, as it can lead to increased regrowth of tumor cells resistant to CTRT and lengthen the overall treatment period [30].

In cervical cancer, NACT has demonstrated complete clinical and pathological response rates ranging between 7.5% and 39% and between 11% and 20%, respectively. These notable response rates may contribute to a decrease in the necessity for adjuvant RT from 53% to 34% [29,31]. Despite its potential, NACT has not consistently translated into improved survival outcomes when compared to surgery alone or definitive RT, and the supporting evidence for its routine use remains limited [29].

Certain studies propose that NACT followed by surgical treatment might enhance survival rates compared to RT alone. However, RT alone without concurrent CT is no longer considered a standard treatment. Administering NACT prior to definitive RT has failed to demonstrate significant benefits, potentially due to the selection of clones resistant to RT. In contrast, this interaction appears less significant between CT and surgery. While the combination of NACT and surgery shows promise, it still lacks robust evidence and remains a practice in several regions globally [32,33].

In 1999, the National Cancer Institute endorsed concurrent CTRT as the standard treatment for locally advanced cervical cancer, citing a 30–50% reduction in mortality [34]. In certain regions, NACT followed by surgery (NCS) has emerged as a potential alternative. Some studies have reported significant advantages over RT alone, particularly in terms of OS and DFS [35]. The most effective assessment of adding CT to surgical treatment involves directly comparing it to surgery alone (Table 2 and Table 3). However, randomized controlled trials [36] and observational studies [37] have yet to establish the efficacy of NCS when comparing to primary surgery. Moreover, recent reviews of NCS for cervical cancer indicate no significant improvement in OS compared to surgery alone [10,31,38].

Moreover, another study has confirmed that NACT followed by surgery significantly prolonged DFS compared with surgery alone (85% vs. 66%, *p* = 0.03) and reduces hospital stay [42]. In 2019, Zhao et al. conducted a meta-analysis to evaluate OS, DFS, and local or distant recurrence in patients treated with NACT followed by surgery compared to surgery alone. Their analysis of 13 studies encompassing 2.158 cervical cancer patients revealed no significant differences between the two groups. However, a subanalysis of eight studies involving 1.544 patients with stages IB2 (at present IB3) to IIB showed that the group receiving NACT prior to radical hysterectomy experienced significantly lower rates of distant metastasis and disease relapse, as well as improved OS. Despite these findings, the meta-analysis did not establish a definitive relation between NACT and prolonged DFS or progression-free survival [43]. As a result, the decision to implement NACT before radical surgery remains contingent on the surgeon’s clinical judgment and expertise. Further research in this area is essential to validate these findings and provide clearer guidance.

Several contemporary prospective and retrospective studies have compared NACT followed by surgery with chemoradiation (Table 4 and Table 5).

Gupta et al. conducted a randomized controlled trial to evaluate NACT using a regimen of paclitaxel and carboplatin followed by radical surgery versus concurrent CTRT in patients with stage IB2, IIA, or IIB squamous cervical cancer. The study demonstrated that concurrent CTRT achieved a higher 5-year DFS rate compared to NACT with surgery, with an absolute difference of 7.4 percentage points favoring CTRT [32]. Stage-specific analysis from the study revealed that for stages IB2 (74% vs. 72.6%) and IIA (74% vs. 72.6%), the differences in 5-year DFS between the NACT plus surgery group and the CTRT group were not statistically significant. However, for stage IIB, the 5-year DFS rate was significantly higher in the CTRT group (79.3%) compared to the NACT plus surgery group (67.2%). No significant difference in OS was observed between the two groups, although the study was not sufficiently powered to draw definitive conclusions on this endpoint. The study was based on the assumption that a taxane-platinum-based NACT regimen would reduce the risk of distant recurrence and improve local control when combined with surgery, as compared to CTRT. However, the findings suggest that adding more CT beyond what is included in concurrent CTRT is unlikely to further enhance survival outcomes in patients with locally advanced cervical cancer. The majority of first recurrences (102 out of 162, or 62.96%) in this trial were local, emphasizing the critical importance of achieving effective local control. In the NACT plus surgery group, surgery was feasible in only 227 patients (72.15%), a proportion similar to that reported in the ongoing European Organization for Research and Treatment of Cancer randomized trial (76% surgical resection rate) [44].

Furthermore, a significant proportion of patients in the surgical group (32.2%) required adjuvant RT or CTRT, resulting in trimodal treatment for a considerable subset of patients [32].

Additionally, and focusing on the subset of IB3-IIA2 cervical cancer patients, a recent study demonstrated that NACT followed by radical surgery was associated with a significant higher DFS rates compared to CTRT; however, the main limitation of the study was the small sample size [30].

A recent meta-analysis that included data from both studies found no significant difference in OS between the treatment groups (HR 1.08; 95% CI 0.86–1.36). However, DFS was in favor of CTRT (HR 1.32; 95% CI 1.07–1.62) [45]. NACT followed by surgery was associated with a 32% higher risk of relapse compared to CTRT, along with a greater incidence of acute toxicity. Although bladder and rectal toxicity were higher in the CTRT group beyond three months, no significant differences were observed at 24 months or later [44]. The only exception was vaginal toxicity, which remained significantly higher in the CTRT group even at 24 months or beyond [32].

Recently, a multicentric Japanese phase II study was carried out to evaluate efficacy and toxicity of neoadjuvant irinotecan and nedaplatin followed by radical hysterectomy and adjuvant CT for locally advanced, bulky uterine cervical cancer [6]. The objective response rate (complete response + partial response) for the NACT regimen was 81.2%. The 2-year and 5-year DFS rates were 87.5% and 78.8%, respectively. The incidence of neutropenia (grade 3/4) during NACT was 6.3% and during adjuvant treatment was 34.4%. This group suggested that this regimen of treatment could improve the prognosis of patients with bulky tumors [6].

Analysis of temporal trends indicates that the proportion of patients undergoing surgery following NACT has remained relatively stable across periods before and after the introduction of standard care protocols (CTRT therapy). Approximately 25–30% of cases continue to be inoperable, necessitating definitive CTRT. Similarly, about 25–30% of patients who receive surgery after NACT require adjuvant radiation or chemoradiation, resulting in trimodal therapy, which is associated with potential toxicity [46].

However, certain patients with stage IB2 and IIA disease may achieve comparable outcomes with NACT followed by surgery, as suggested by subgroup analyses in the Gupta et al. study [32,46]. Also, the hypothesis that NACT addresses macrometastasis disease and enhances distant control has also not been substantiated in recent clinical trials, as reflected in OS outcomes [46].

Current evidence strongly supports concurrent CTRT as the standard treatment for bulky cervical tumors, while it is possible that specific subsets of patients—defined by factors such as age, disease stage, and tumor size—may benefit from NACT followed by surgery in terms of equivalent oncological outcomes and improvements in areas like sexual health [46].

### 2.3. Fertility Preservation After Neoadjuvant Chemotherapy in Bulky Tumors

According to data from the Global Cancer Observatory, cervical cancer ranks as the third most common cancer and the second leading cause of cancer-related death among women under the age of 45 worldwide, corresponding up to 40% of all cervical cancers [47,48]. This underscores the fact that many patients have not yet fulfilled their reproductive desires at the time of cervical cancer diagnosis, making it crucial to determine whether they are candidates for fertility-sparing treatment.

Currently, for women with reproductive desire, the international guidelines recommend fertility preservation for stage IB1 or lower according to 2018 FIGO classification of SCC or Human-Papillomavirus-ADC, always after excluding lymph node involvement, as it has shown comparable survival rates to non-uterine-preserving surgery [49]. The goal of FSS is to achieve tumor resection with adequate clear margins while preserving the upper portion of the cervix. This can be achieved through cervical conization or simple trachelectomy for stages IA1 and IA2, simple trachelectomy for stage IB1 without LVSI, and type B radical trachelectomy for stage IB1 with LVSI [2].

FSS in patients with tumor sizes ≥ 2 cm is associated with a higher recurrence rate and is, therefore, not considered a standard treatment [50,51]. Additionally, these patients are more likely to require adjuvant RT after surgery, which significantly reduces the success rate of fertility sparing treatment. Furthermore, an extensive surgical resection may lead to a higher incidence of operative complications and a decreased pregnancy rate [47]. However, some studies have evaluated the feasibility of fertility preservation in tumors larger than 2 cm following the administration of NACT, as NACT can reduce the tumor volume, thus increasing the proportion of patients who are suitable for FSS [31]. Additionally, tumor regression allows for a less extensive surgical approach, which can subsequently improve fertility rates [50,52].

Some studies have reported favorable survival and pregnancy outcomes following NACT in tumors measuring between 2 and 4 cm [53,54,55]. In this regard, robust evidence on oncological and obstetric outcomes is expected in 2025 based on the results of the CONTESSA trial [56]. In this study, patients with stage IB2 who wish to preserve fertility will receive NACT, and, in the case of a complete or partial response (tumor size < 2 cm), they will undergo fertility-sparing surgery.

Regarding bulky cervical tumors, few studies have explored the feasibility of FSS, with this approach being considered experimental. Most of the available evidence consist of case reports and case series of patients with bulky tumors who underwent FSS after an adequate response to CT, showing low recurrence and mortality rates [57,58,59,60,61] (Table 6). The most robust evidence comes from a systematic review conducted by Viveros-Carreño et al. in 2022, which analyzed data from 11 studies investigating FSS after NACT in patients with SCC, ADC, or ADSC, FIGO 2018 IB3 stage cervical tumors [8]. Of the 40 patients included, FSS was successfully achieved in 26 (65%). This small sample size underscores the limited evidence available regarding FSS in bulky tumors. The median tumor size in this study was 4.6 cm, and all patients underwent platinum-based chemotherapy, achieving a complete pathological response in 56% of cases. None of the patients with complete response had a recurrence. Survival outcomes were notably high, with a 4.5-year DFS rate of 92.3% and an OS rate of 100%. In terms of pregnancy outcomes, 67% (four out of six) of patients who attempted conception were successful, although a high incidence (60%) of preterm delivery was observed following radical trachelectomy.

Another systematic review performed by Di Donato et al. included 48 women affected by stage I cervical cancer ≥ 4 cm who underwent FSS [62]. In this case, only three patients (6.3%) experienced a recurrence and one of them (2.1%) died of disease. Similar to the systematic review from Viveros-Carreño et al. [8], the reported 5-year DFS and OS rates were over 90% (92.4% and 97.6%, respectively). Pregnancy outcomes were also favorable, with an estimated pregnancy rate of 80%, a live birth rate of 83.3%, and a premature delivery rate of 20% [62].

Despite the findings reported in these two systematic reviews, the lack of high-quality studies remains a significant limitation. Currently, no randomized trials are available, and most existing studies are retrospective, with different inclusion and exclusion criteria, resulting in heterogeneous cohort populations. These limitations hinder the ability to draw definitive conclusions. Further studies are necessary to clarify the role of FSS in patients with bulky tumors responding to NACT.

**Table 6:** Literature review of patient characteristics and survival outcomes for bulky cervical cancer tumors treated with NACT plus fertility-sparing surgery.

Author/Year	Maximum Tumor Size (cm)	NACT (nº Cycles)	Clinical/ Radiological Response	Pathological Response	Type of Surgery	Follow Up (Months)	Recurrence	Death
Palaia, 2011 [57]	5.5	3 cycles TAX + CIS + IFO	Complete	Complete	ST	18	No	No
Vercellino, 2012 [58]	4.2	2 cycles TAX + CIS	Partial	Partial	VRT	70	No	No
	4.2	2 cycles TAX + CIS + IFO	Partial	Partial	VRT	15	No	No
Hamed, 2012 [60]	6.0	4 cycles TAX + CIS	Complete	Complete	RRT	16	No	No
Tsubamoto, 2012 [59]	4,3	3 cycles CIS + Nedapalin	Complete	Complete	ST	86	No	No
	6.0	3 cycles CIS + Irinotecan	Complete	Complete	ST	120	No	No
Van Gent, 2014 [61]	4.2	6 cycles TAX + CIS	N/R	Partial	ART	6	No	No
Lanowska, 2014 [63]	5.0	3 cycles TAX + CIS + IFO	N/R	Complete	VRT	79	No	No
	4.2	2 cycles TAX + CIS + IFO	N/R	Complete	VRT	6	No	No
Rovoba, 2014 [64]	4.2	3 cycles CIS + IFO	N/R	Complete	ST	71	No	No
	4.3	3 cycles CIS + IFO	N/R	Partial	ST	62	Yes	No
Hauerberg, 2015 [65]	4.5	CIS + IFO-5-Fluorouracil	N/R	N/R	VRT	68	No	No
Feng, 2016 [66]	6.6	3 cycles TAX + CIS	Partial	Complete	Conization	72	No	No
Slama, 2016 [67]	4.2	3 cycles CIS + IFO	Complete	Partial	ST	104	No	No
	5.9	3 cycles CIS + IFO	Partial	Partial	Conization	77	Yes	No
Marchiole, 2018 [68]	4.2	4 cycles TAX + CIS + IFO	Partial	Partial	LRVT	100	No	No
	4.5	3 cycles TAX + CIS + Epirrubicin	Complete	Complete	LRVT	84	No	No
	4.5	3 cycles TAX + CIS + Epirrubicin	Partial	Partial	LRVT	47	No	No
	4.4	4 cycles TAX + CIS	Complete	Complete	LRVT	6	No	No
Tesfai, 2019 [69]	5.0	5 cycles TAX + CIS	Partial	Complete	ART	65	No	No
	4.5	6 cycles TAX + CIS	Partial	Complete	ART	26	No	No
	5.0	3 cycles TAX + CIS	Complete	Complete	ART	45	No	No
	5.0	6 cycles TAX + CIS	Complete	Complete	ART	64	No	No
Rendón, 2021 [70]	5.0	3 cycles TAX + CARBO	Partial	Partial	ART	48	No	No
	4.5	3 cycles TAX + CARBO	Partial	Partial	LRT	42	No	No
	5.0	3 cycles TAX + CARBO	Complete	Complete	Conization	96	No	No
	4.3	3 cycles TAX + CIS + IFO	Partial	Partial	LRT	46	No	No
	4.1	3 cycles TAX + CIS + 5-Fluouracil	Partial	Complete	ART	119	No	No
	4.3	6 cycles TAX + CARBO	Complete	Complete	LRT	45	No	No

NACT: neoadjuvant chemotherapy; ST: simple trachelectomy; VRT: vaginal radical trachelectomy; RRT: robotic-assisted radical trachelectomy; ART: abdominal radical trachelectomy; LRVT: laparoscopic-assisted vaginal radical trachelectomy; LRT: laparoscopic radical trachelectomy; TAX: paclitaxel; CIS: cisplatin; IFO: Ifosfamide; CARBO: carboplatin; N/R: not reported.

### 2.4. Adjuvant Radiotherapy in Bulky Tumors

In 1999, Sedlis et al. published the results of a prospective randomized trial designed to evaluate the benefit of adjuvant RT after radical hysterectomy in patients with intermediate-risk factors for recurrence identified in the pathological analysis of the surgical specimen: tumor size > 4 cm, deep stromal invasion, and presence of LVSI [5]. Their results demonstrated that the administration of RT in patients with a combination of these factors was associated with a 47% reduction of the recurrence risk. Since then, this has been the standard management recommended by most scientific societies [2,4]. In recent years, however, doubts have arisen about the applicability of the Sedlis study’s findings to current cervical cancer management. This is because the characteristics of the patients in the Sedlis cohort differ from those currently undergoing radical hysterectomy. On one hand, tumor size was assessed clinically rather than using pelvic MRI, which is the current recommended method [71]. On the other hand, thanks to the development of the sentinel lymph node biopsy technique with ultrastaging, a higher number of patients with lymph node involvement are now being identified [72,73]. These patients, who are considered to be at high risk of recurrence, would currently receive adjuvant chemotherapy in addition to RT, but they were not identified in the Sedlis cohort, so were classified as intermediate-risk, rather than high-risk recurrence patients, which may have influenced the study’s survival results [2,74]. Lastly, the Sedlis study does not describe the type of parametrial resection performed during the radical hysterectomy procedure. Thus, it is impossible to determine whether the surgical technique employed was appropriate for each case, as for patients with large tumor sizes, a type C2 parametrectomy according to the Querleu–Morrow classification would be indicated [2].

In bulky tumors, this controversy is particularly relevant, as these patients inherently have an intermediate risk factor for recurrence and, therefore, a higher likelihood of receiving postoperative RT if Sedlis criteria are followed, this being one of the main reasons why these patients are usually treated with concurrent CTRT instead of surgery.

Since the publication of the Sedlis trial results, few studies have compared the differences between adjuvant RT and observation in patients presenting a combination of intermediate-risk factors. Most of these studies are retrospective with a limited number of cases. Although the findings are contradictory, the majority do not demonstrate a clear benefit of adjuvant RT in survival rates [75].

In this context, van der Velden reported a 15.5% relapse rate in a cohort of 161 patients diagnosed with intermediate-risk cervical cancer who did not receive adjuvant RT following type C2 radical hysterectomy. Only 2.5% of the patients died from disease after developing a loco-regional recurrence [76]. Similarly, Cibula et al. reported a 95.7% 5-year DFS rate in patients who met two intermediate-risk factors undergoing observation after radical hysterectomy, with only 1.6% local recurrence rate [77]. After these results were observed, they developed a subanalysis of the intermediate-risk patients from the cohort of the SCANN (international multicenter Surveillance in Cervical CANcer) study, analyzing almost 700 patients. The 5-year DFS (*p* = 0.365) and OS (*p* = 0.281) were not significantly different between the no adjuvant treatment and adjuvant treatment groups. Moreover, in a subgroup analysis of patients with tumor size ≥ 4 cm, adjuvant treatment was not associated with survival benefit (*p* = 0.129 for DFS; *p* = 0.575 for OS) [78]. The authors attributed the substantially better outcomes observed in these studied compared with the Sedlis trial to more accurate pre-operative and pathological staging, as well as improvement in surgical technique. In the same line, the retrospective study conducted by Ishizawa et al. did not identify significant differences between the two groups in the subgroup analysis stratified by tumor size [79]. However, adjuvant RT was associated with a higher incidence of side effects. Furthermore, an analysis of one of the largest cohorts to date, based on the National Cancer Database, found no improvement in OS among patients who received adjuvant RT (with or without CT). DFS could not be evaluated in this study, as data on recurrences were not collected [80]. The study conducted by Tuscharoenporn et al. also found no differences in OS or DFS between the two groups. However, the pelvic recurrence rate was lower in the group with two or more intermediate risk factors that received RT [81].

Other retrospective studies that compared survival differences based on the number of Sedlis criteria that patients met also found no differences between the RT group and the observation group, regardless of whether one, two, or three intermediate-risk factors were present [82,83].

The strongest evidence available to date comes from two metanalysis, although their results are contradictory. It is important to note that the studies included in these metanalysis are mostly retrospective and exhibit significant heterogeneity regarding the number and the definition of the Sedlis criteria included, which limits the comparability of their results. In the one published in 2019 by Sagi-Dain et al., which included five retrospective studies, adjuvant RT was associated with a significant reduction in the risk of recurrence in patients meeting two intermediate-risk factors (OR 1.86, *p* = 0.04). However, this benefit was no longer significant when only patients with a single intermediate-risk factor were analyzed, and no significant differences in OS between the two groups were found (*p* = 0.08) [84].

More recent data are available from the metanalysis conducted by Gómez-Hidalgo et al. in 2022. In the data obtained from the eight studies included, Sedlis study among them, no differences were found in OS (*p* = 0.06) or DFS (*p* = 0.197) between the adjuvant RT group and the no further treatment group [85].

According to these findings, the benefit of adjuvant RT following radical hysterectomy in patients with at least two intermediate-risk recurrence factors remains unclear. However, data from prospective randomized studies comparing both strategies are needed. In this line, an international multicenter randomized non-inferiority study between observation and adjuvant treatment (CERVANTES trial) has been developed in patients with intermediate-risk factors after radical hysterectomy. Patients will be randomized to the additional treatment arm or to the adjuvant treatment arm with external beam RT ± BT ± concomitant chemotherapy. Its results, anticipated by 2031, are expected to provide the definitive evidence required to establish the most appropriate management strategy for these patients [86]. If it is confirmed that patients with a combination of intermediate recurrence risk factors do not require adjuvant treatment, those with tumor sizes ≥ 4 cm at diagnosis could become better candidates for surgical treatment. This approach would lower the likelihood of requiring RT and, consequently, reduce the probability of combined treatment modalities.

## 3. Summary and Conclusions

The optimal treatment strategy for bulky cervical tumors remains a matter of debate due to limited high-quality evidence and significant variability in clinical outcomes based on tumor characteristics and treatment modalities. While radical surgery offers several advantages compared to concurrent CTRT, including precise staging, reduced pelvic recurrence, and better quality of life compared to RT, its outcomes are influenced by other factors such as histology and lymph node involvement. Additionally, the likelihood of requiring adjuvant treatment after surgery must be considered, as this may lead to an increased risk of adverse effects. Patients with AC or ASC histologies may benefit more from radical surgery than from concurrent CTRT due to the radioresistant nature of these subtypes.

NACT followed by surgery cannot yet be regarded as the standard of care for patients with bulky cervical tumors. This treatment strategy requires additional clinical research to achieve strong, high-quality evidence, particularly for groups of patients who might derive potential benefits in terms of survival, reduced toxicity, and improved quality of life when compared to the established gold-standard treatment of concurrent CTRT. Similarly, the evidence supporting FSS following NACT in bulky tumors remains scarce. If performed in the context of experimental studies, radical trachelectomy after complete or partial response should be the technique of choice.

Adjuvant RT after radical hysterectomy in patients with bulky tumors who present other intermediate risk factors for recurrence is currently a matter of debate, particularly when proper preoperative patient selection and adequate parametrial resection have been performed. The results of prospective randomized trials will provide definitive evidence on this topic.

This narrative review has inherent limitations, including the potential for selection bias and the lack of a standardized methodology for article inclusion, compared to a systematic review. Additionally, the limited available evidence on this topic, often based on small or heterogeneous studies, restricts the ability to draw definitive conclusions. However, despite these limitations, we have provided an updated summary of the available evidence on a topic that remains poorly studied, offering valuable insights that may contribute to a better understanding and management of bulky cervical tumors. Further high-quality research is needed to establish more robust clinical guidelines.

## Figures and Tables

**Table 1:** Literature review of radical surgery outcomes as a treatment for bulky cervical cancer.

Author/Year	N	Stage (FIGO 2009)	5-Year OS (%)	5-Year DFS (%)	LRR (%)	DM (%)
Landoni, 1997 [26]	55	IB2/IIA2	70	63	20	15
Huang, 2012 [23]	60	IB2/IIA2	NA	91	NA	NA
Rungrugang, 2012 [25]	401	IB2	83	87	NA	NA
Liu, 2020 [24]	235	IB2/IIA2	81.5	73	NA	NA
Zhou, 2022 [27]	975	IB2/IIA2	85.9	80.8	NA	NA
Chen, 2023 [9]	Entire cohort: 227 Low risk: 99 High risk: 128	IB2/IIA2 IB2/IIA2 IB2/IIA2	84 86 83	79 84 75	10 8 12	14 9 18

OS: overall survival; DFS: disease-free survival; LRR: locoregional recurrence; DM: distant metastases; NA: not assessable.

**Table 2:** Literature review of treatment outcomes for bulky cervical cancer tumors: NACT plus surgery vs. primary radical surgery.

Author/Year	Treatment	N	FIGO stage (2009)	Study Design	OS (%)	*p*	DFS (%)	*p*
Hong, 2006 [36]	NACT/RS	52/54	IB	RCT	85/76 (5 y)	<0.05	83/74 (5 y)	<0.05
Behtash, 2006 [38]	NACT/RS	22/160	IB-IIA	Retrospective	28/68 (5 y)	NS	41/30 (10 y)	NS
Chen, 2008 [39]	NACT/RS	72/70	IB2-IIB	RCT	71/58 (4 y)	NS	NACT improved DFS	<0.05
Cho, 2009 [40]	NACT/RS	51/35	IB2-IIA	Retrospective	93/91 (5 y)	NS	93/81 (5 y)	NS
Kim, 2010 [37]	NACT/RS	61/183	IB1-IIA	Matched case	RS improved OS in stage IIA	-	-	NS
Lee, 2011 [41]	NACT/RS	33/41	IB2-IIA	Retrospective	-	-	90/81 (5 y)	NS
Qin, 2016 [42]	NACT/RS	30/35	IIA2-IIB	Retrospective cohort	80/71 (3 y) NS	NS	85/66 (3 y)	<0.05

FIGO: International Federation of Gynecology and Obstetrics; OS: overall survival; DFS: disease free-survival; NACT: neoadjuvant chemotherapy; RS: primary radical surgery; RCT: randomized controlled trial; NS: not significant.

**Table 3:** Literature review of NACT regimens for bulky cervical tumors.

Author/Year	NACT
Hong, 2006 [36]	75 mg/m^2^ IV Cisplatin (Day 1) + IV 5-Fuorouracil 24 mg/kg/d (Day 1 to 5) (2 cycles)/21 days
Behtash, 2006 [38]	50 mg/m^2^ IV Cisplatin + 1 mg/m^2^ IV Vincristine (3 cycles)/10 days
Chen, 2008 [39]	100 mg/m^2^ IV Cisplatin (Day 1) + 4 mg/m^2^ IM Mitomycin C (Day 1 to 5) + IV 5-Fluorouracil 24 mg/kg/day (Day 1 to 5) (2 cycles)/14 days
Cho, 2009 [40]	135 mg/m^2^ IV Paclitaxel + 75 mg/m^2^ IV Cisplatin or IV Carboplatin (AUC 5) (2 cycles)/21 days
Kim,2010 [37]	175 mg/m^2^ IV Paclitaxel + IV Carboplatin (AUC 5) + IV 5-flourouracil (1000 mg/m^2^ for 5 consecutive days) and IV Cisplatin (60 mg/m^2^) + IV 5-fluorouracil (1000 mg/m^2^ for 5 consecutive days) and IV Carboplatin (AUC 5) (2–3 cycles)/21 days
Lee, 2011 [41]	175 mg/m^2^ IV Paclitaxel + IV Carboplatin (AUC 5) (2–3 cycles)/21 days
Quin, 2016 [42]	175 mg/m^2^ IV Paclitaxel + 75 mg/m^2^ IV Cisplatin (2–3 cycles)/21 days

IV: intravenous; IM: intramuscular; AUC: area under curve.

**Table 4:** Literature review of treatment outcomes for bulky cervical cancer tumors: NACT plus surgery vs. chemoradiotherapy.

Author/Year	Treatment	N	FIGO Stage (2009)	Study Design	OS (%)	*p*	DFS (%)	*p*
Gupta, 2018 [32]	NACT/CTRT	316/317	IB2-IIB	RCT	75.4/74.7 (5y)	NS	69/77 (5y)	<0.05
Akhavan, 2021 [30]	NACT/CTRT	46/51	IB3-IIA2 *	Retrospective	97/90 (3y)	<0.05	88/66 (3y)	<0.05
Kenter, 2023 [44]	NACT/CTRT	314/312	IB2-IIB	RCT	72/76 (5y)	NS	57/65.6 (5y)	<0.05

FIGO: International Federation of Gynecology and Obstetrics; OS: overall survival; DFS: disease-free survival; NACT: neoadjuvant chemotherapy; CTRT: chemoradiotherapy; RCT: randomized controlled trial; NS: not significant. * FIGO 2018.

**Table 5:** Literature review of NACT plus CTRT regimens for bulky cervical tumors.

Author/Year	Treatment
Gupta, 2018 [32]	IV Paclitaxel (175 mg/m^2^) + IV Carboplatin (3 cycles/21 days))/EBRT (40 Gy/20 fractions) + BT (30 Gy/2 fractions or 7 Gy/5 fractions).
Akhavan, 2021 [30]	IV Cisplatin 80 mg/m^2^ + IV Paclitaxel 60 mg (3 cycles/10 days)/EBRT (45 Gy) + 40 mg/m^2^ Cisplatin/7 days + BT (30 to 40 Gy).
Kenter, 2023 [44]	IV Cisplatin (75 mg/m^2^. 3 cycles/21 days)/IV Cisplatin (40 mg/m^2^. 5–6 cycles/7 days) + EBRT (50 Gy) + BT.

IV: intravenous; EBRT: external beam radiation therapy; BT: brachitherapy.
